# Using exergame-based exercise to prevent and postpone the loss of muscle mass, muscle strength, cognition, and functional performance among elders in rural long-term care facilities: A protocol for a randomized controlled trial

**DOI:** 10.3389/fmed.2022.1071409

**Published:** 2022-12-13

**Authors:** Sheng-Hui Tuan, Ling-Hui Chang, Shu-Fen Sun, Ko-Long Lin, Yi-Ju Tsai

**Affiliations:** ^1^Institute of Allied Health Sciences, College of Medicine, National Cheng Kung University, Tainan City, Taiwan; ^2^Department of Rehabilitation Medicine, Cishan Hospital, Ministry of Health and Welfare, Kaohsiung City, Taiwan; ^3^Department of Occupational Therapy, College of Medicine, National Cheng Kung University, Tainan City, Taiwan; ^4^Department of Physical Medicine and Rehabilitation, Kaohsiung Veterans General Hospital, Kaohsiung city, Taiwan; ^5^School of Medicine, College of Medicine, National Yang Ming Chiao Tung University, Taipei City, Taiwan; ^6^School of Medicine, Kaohsiung Medical University, Kaohsiung City, Taiwan; ^7^Department of Physical Therapy, College of Medicine, National Cheng Kung University, Tainan City, Taiwan

**Keywords:** exergame, sarcopenia, frailty, long term care, multicomponent training, RingFit Adventure

## Abstract

**Objective:**

Elderly individuals in long-term care facilities (LTCFs) have a higher prevalence of sarcopenia than those in the community. Exercise is the gold standard for preventing and treating sarcopenia. Regarding exercise, multicomponent exercises, including progressive resistance training (PRT), are beneficial. However, developing routine, structured exercise programs for the elderly in LTCFs is difficult because of a shortage of healthcare providers, particularly in rural regions. Exergame-based exercises can increase a player’s motivation and reduce staff time for an intervention. Nintendo Switch RingFit Adventure (RFA) is a novel exergame that combines resistance, aerobic, and balance exercises. In this study, we aim to investigate the clinical effectiveness of RFA on muscle and functional performance parameters among the elderly in LTCFs.

**Methods:**

The EXPPLORE (using **EX**ergame to **P**revent and **P**ostpone the **LO**ss of muscle mass, muscle strength, and functional performance in **R**ural **E**lders) trial is a single-center randomized controlled trial involving elderly individuals (≥60 years) living in LTCFs in rural southern Taiwan. The participants will be equally randomized to the intervention group (exergame-based exercise plus standard care) or the control group (standard care alone). Both groups will receive standard care except that the intervention group will receive exergame-based exercises at the time previously scheduled for sedentary activities in the LTCFs. The exergame-based exercise will be performed using RFA in the sitting position with a specialized design, including arm fit skills and knee assist mode. Each session of the exercise lasts 30 mins and will be performed two times per week for 12 weeks. The primary outcomes will be the osteoporotic fracture index, appendicular skeletal muscle mass index, dominant handgrip strength, and gait speed. Meanwhile, the secondary outcomes will be the dexterity and agility, muscle strength and thickness, range of motion of the joints of the dominant upper extremity, Kihon checklist, Medical Outcomes Study 36-Item Short-Form Health Survey, and Brain Health Test.

**Discussion:**

This trial will provide valuable knowledge on whether exergames using RFA can counteract physical decline and improve quality of life and cognition among the elderly in LTCFs.

**Clinical trial registration:**

[www.ClinicalTrials.gov], identifier [NCT05360667].

## Background

Taiwan is now an aging society, and the population of elderly individuals aged ≥65 years constituted 15.95% of the total population in 2020. It is estimated that Taiwan will become a super-aged society as the number of individuals aged ≥65 years grow to be more than 20% of the total population by 2025 (1). Aging and inactivity are associated with declines in muscle mass, architecture, and strength (2, 3). The rate of muscle loss ranges from 1 to 2% per year for individuals more than 50 years old (4). Sarcopenia, defined as an age-related loss of skeletal muscle mass and a decline in muscle strength and physical performance (5), is thus emerging as an important issue in modern society. As reported by the Asian Working Group for Sarcopenia (AWGS), the prevalence of sarcopenia in community-dwelling older men and women in Taiwan was 9.3 and 4.1%, respectively (6). For mobility-impaired older adults, regular physical activity can prevent further disabilities and improve their overall health (7, 8), while physical inactivity is an important factor contributing to the development of sarcopenia (9). The prevalence of sarcopenia is much higher in individuals living in care facilities than those residing in the community, ranging between 17.7 and 73.3% in long-term nursing homes (10) and between 22 and 87.1% in daycare centers, because they are older and sicker and require more assistance with their activities of daily living (ADLs) after being admitted to care facilities (10–12).

The first-line strategies for preventing and treating sarcopenia focus on preserving skeletal muscle mass and maintaining muscle strength (13). A newly published network meta-analysis revealed that mixed exercises and physical activity with nutritional supplementation are the most effective interventions for sarcopenia (14). Resistance training (RT), particularly progressive RT (PRT) (15, 16), and multicomponent exercises have moderate- to high-quality evidence for their positive and significant effects on increasing muscle mass, muscle strength, and physical performance among elderly individuals with sarcopenia (17, 18). Moreover, some studies showed that low-intensity RT [≤50% 1 repetition maximum (RM)] is sufficient to induce strength gains (19, 20). However, the optimal exercise prescription, regarding intensity, type, frequency, duration, and progression, for preventing and treating sarcopenia among elderly individuals is still controversial (14, 17).

Taiwan is one of the fastest-aging countries in the world (21). The Taiwanese government established a long-term healthcare system in 2010, which was reformed into The Long-Term Care 2.0 Plan (LTC 2.0) in 2017 to guarantee suitable services in response to the fast-growing care needs of older individuals (21). The LTC 2.0 aims to provide aging-in-place values into practice by delivering integrated home- or community-based primary healthcare and preventive services (22). Based on the consensus of the AWGS in 2019, community-based care facilities are important places for integrated LTC services to prevent or delay disability, particularly for individuals who are physically inactive or at risk of sarcopenia (23). These settings are essential, particularly in rural regions in Taiwan, where the proportion of elderly individuals is higher than that in urban areas (24) and the healthcare resources are limited (25).

The principles of PRT and multicomponent exercise programs include regular, mass-practiced, mildly overwhelming engagement (26). To achieve these principles, the devotion of time, workforce, and money are critical. Staffing constraints and resource shortages have made it challenging to promote regular exercise programs in long-term care facilities (LTCFs) (27, 28). Exergames are defined as any type of video game that requires the movement of the player’s entire body, allowing real-time interaction (29). Exergames breakdown the barriers of repetitive and monotonous physical exercises since they contain attractive and multisensory game environments with an immersive environment in which the interaction occurs through whole-body movements (30). Moreover, the gamified approach and immersive scenarios motivate older individuals to acquire a greater commitment to the practice of physical and rehabilitative exercises (31). Therefore, playing exergames reduces staff time for intervention, encourages patients to perform relatively high-energy movements, and increases their motivation. Among community-dwelling older individuals with or without specific diseases (32), studies have proved the therapeutic application of exergames in improving lower limb strength (33), gait speed (34), balance (35), and cognitive function (34, 36). Few studies have evaluated the clinical effectiveness of exergames in LTCFs, and most outcomes of these studies pertained to health-related quality of life (HRQoL), cognition, and general functional status (37–39). Our team found that the handgrip strength (HGS) and walking speed of elderly individuals with sarcopenia living in care facilities improved significantly after 12 weeks of exergame-based progressive RT(40). However, it was a quasi-experimental study involving relatively few participants, and the platform to deliver exergames was difficult for older adults to use. Therefore, in this study, we will evaluate the feasibility and clinical application of a novel exergame-based multicomponent training program using Nintendo Switch^®^ RingFit Adventure (RFA), which could deliver optimal exercise intensity for each player and perform fine-tuned upregulation and downregulation based on performance after each game, among elderly individuals living in rural care facilities.

## Methods and analysis

### Trial design and ethics

**FIGURE 1:**
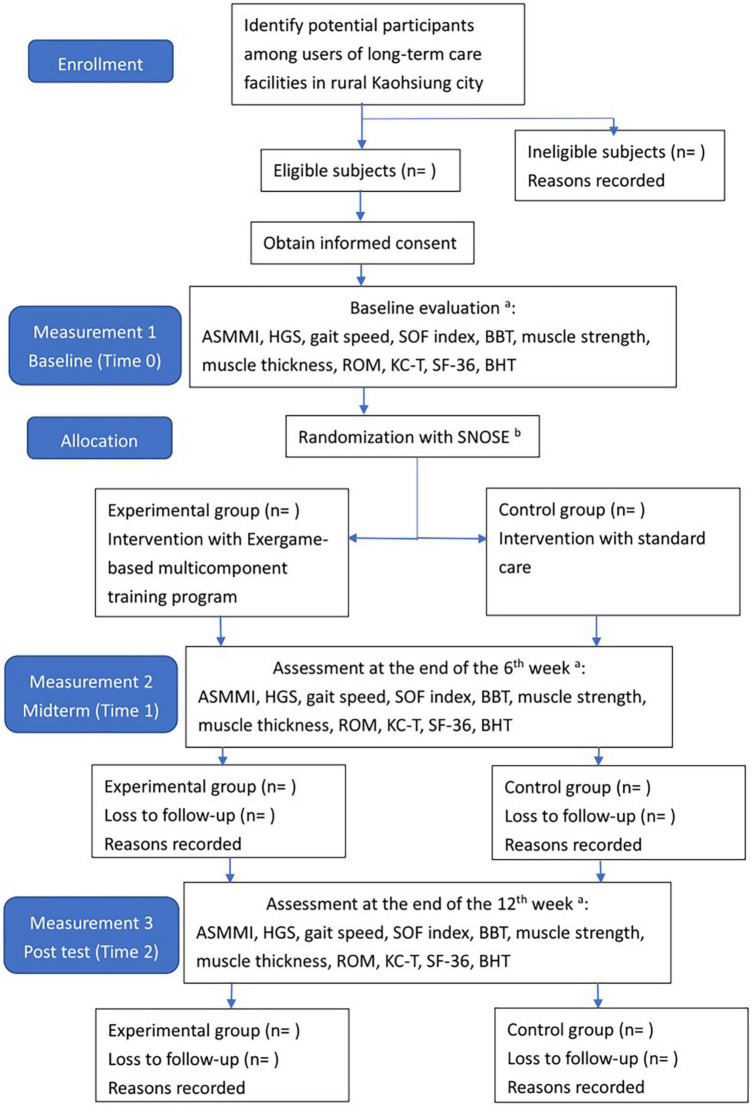
The flowchart of the participant inclusion and data collection process. ^a^See Table 1; ^b^sequentially numbered, opaque, and sealed envelopes. ASMMI, appendicular skeletal muscle mass index; HGS, handgrip strength; SOF index, study of osteoporosis index; BBT, box and block test; ROM, range of motion; KC-T, Kihon checklist Taiwan version; SF-36, short form 36 questionnaire; BHT, brain health test.

This randomized controlled trial (RCT) compares an exergame-based multicomponent exercise training (exergame-based exercise) to the standard care in older users of LTCFs in rural regions. The trial design adheres to the Standard Protocol Items: Recommendations for Interventional Trials (SPIRIT) guidelines for RCT (41) and the Consolidation Standards of Reporting Trials guidelines (42). The flowchart of this study is shown in Figure 1. This study will be conducted according to the Declaration of Helsinki, was approved by the Institutional Review Board of a medical center in Taiwan (approval number: B-ER-111-058), and was registered at www.ClinicalTrials.gov (registration number: NCT05360667).

**TABLE 1:** The flowchart of exergame-based multicomponent training program.

Timeframe	Activity	Activity description	Mainly target muscles and joints
0–10 min	Warm-up	Flexibility exercise.	Progressive static stretch of the neck, chest, arm, thighs, and legs.
11–50 min	Main training	Ringfit Adventure with knee assist mode. The player controls the character in the Adventure Mode by squeezing and stretching the Ring-Con.	**Muscles:** trapezius, triceps, pectoralis major and minor, and core muscles. **Joints:** shoulder horizontal adduction/abduction, shoulder external/internal rotation, and elbow flexion
		To defeat the monsters in each stage, the players has to use the combination of the following six arm fit skills.	Not applicable
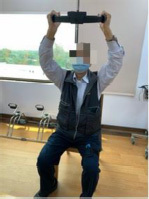		**Overhead press:** Hold the Ring-Con overhead and squeeze it.	**Muscles:** deltoid, and biceps brachii **Joints:** shoulder flexion, and elbow flexion/extension
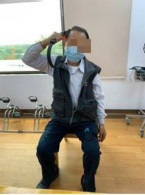		**Shoulder press:** Hold the Ring-Con on each side of the shoulder and squeeze it.	**Muscles:** biceps/triceps brachii **Joints:** elbow flexion/extension
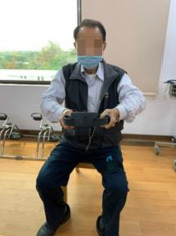		**Front press:** Hold the Ring-Con below umbilicus with anterior-tilting of the trunk slightly.	**Muscles:** trapezius, triceps, pectoralis major and minor, and core muscles. **Joints:** shoulder horizontal adduction/abduction, and elbow flexion
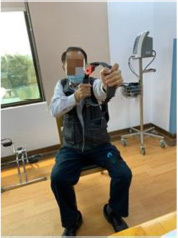		**Bow pull:** Put the Ring-Con in front of the chest and pull it like it is a bow.	**Muscles:** biceps/triceps brachii, and latissimus dorsi **Joints:** shoulder adduction/abduction, elbow flexion/flexion, and wrist flexion/extension
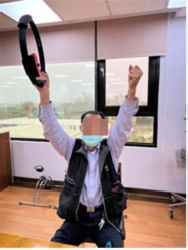		**Overhead arm spin:** raise both arms straight up and twist the wrists.	**Muscles:** deltoid and shoulder girdles **Joints:** shoulder flexion, elbow pronation/supination, and wrist flexion
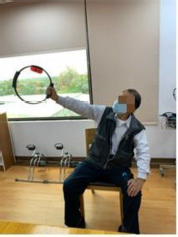		**Tricep kickback:** With elbow locked, move the Ring-Con up-and-down	**Muscles:** triceps, and spinal erectors **Joints:** shoulder horizontal adduction/abduction, and elbow flexion/extension
51–60 min	Cool-down	Flexibility exercise.	Progressive static stretch of the neck, chest, arm, thighs, and legs.

### Selection of participants

All individuals, who participate regularly in LTCFs, including daycare centers and nursing homes, in rural regions of Kaohsiung City, southern Taiwan, will be eligible for study participation. The inclusion criteria are as follows: individuals (a) aged ≥60 years, (b) those living or participating in LTCFs for at least 1 month, (c) those who can understand and speak Chinese or Taiwanese, (d) those with sufficient cognitive capacity (judged by the researchers) to give informed consent and participate in the exergame-based exercise and data collection, and (e) those who can sit for more than 50 min for training and can complete the measurement of gait speed. (a) Individuals who have significant cardiopulmonary diseases, (b) those who regularly receive oxygen supplementation, (c) those who have uncontrollable hypertension, and (d) those who had a recent infection or fracture or were diagnosed with other diseases that might prohibit them from participating in exercises according to the guidelines of the American College of Sports Medicine will be excluded from this study (43). Participants in the intervention group will receive standard care with the exergame-based exercise for 12 weeks, whereas those in the control group will receive standard care routinely applied in LTCFs as usual. We will replace the scheduled sedentary activities in the LTCFs, such as singing, table games, and gardening, with the exergame-based exercise in the intervention group. Meanwhile, participants in the control group will perform the aforementioned sedentary activities in the LTCFs as usual. Therefore, the time for activities in the two groups will be the same. In addition to the exergame-based exercise and standard care, all participants will perform the usual traditional rehabilitation provided by therapists at the LTCFs.

### Randomization

We will use sequentially numbered, opaque, sealed envelopes containing a group assignment number created by a person who is not clinically involved and, therefore, could be blinded to this study. Participants who fulfilled the inclusion and exclusion criteria will be equally randomized to the intervention and control groups with an allocation ratio of 1:1.

### Exergame-based multicomponent training program (intervention group)

**FIGURE 2:**
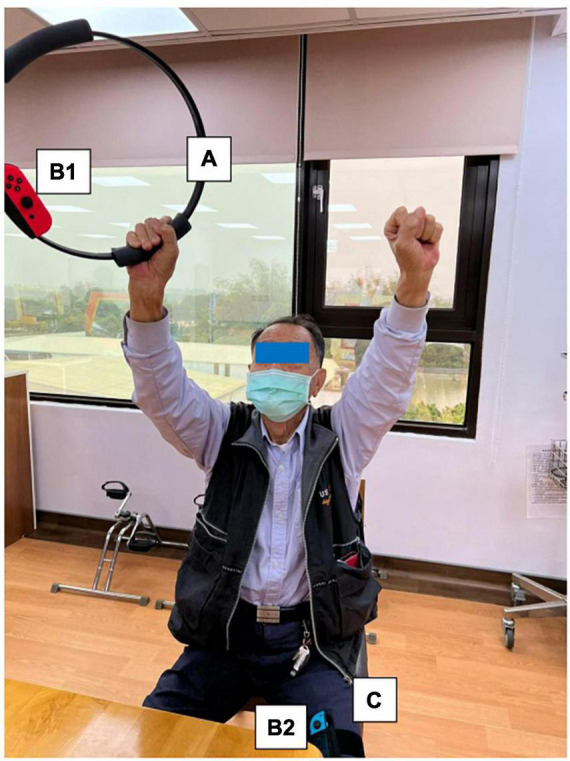
Components of Nintendo Switch. To play the RingFit Adventure, the player needs to use the Nintendo Switch, which includes the following devices: A. Ring-Con: A Pilates ring the user holds; B. Joy-Con: A wireless controller. One of the Joy-Con (B1) should be placed on the Ring-Con (A). The other Joy-Con (B2) should be affixed to a Leg Strap (C) on the thigh of the player.

The program consists of PRT and functional movement of the four extremities but mainly the upper limbs. We will use RFA to deliver the program. RFA is a Nintendo Switch^®^-based exergame. It requires one Nintendo Switch video game console, one Ring-Con (a Pilates ring that the user holds), one wireless controller Joy-Con (one should be placed on the Ring-Con and the other one should be affixed into a leg strap on the thigh of the player), and one display screen (Figure 2). The exergame-based exercise will be performed two times per week, at least 48 h apart from each training session, 50 min per session (10 min each for a warm-up and cool-down and 30 min for the main program), for 12 weeks, and will be supervised by a therapist.

RingFit Adventure is a fitness action role-playing game. The player advances the story while exercising as the movement of the player is linked to the main character on the screen. The movements of the player and battle actions are based on the performance of certain physical activities using the Ring-Con and leg strap (44). Ring-Con is a controller of the system with a built-in high-precision force and strain sensor that detects and digitizes the player’s movements, such as stretching and squeezing. Joy-Con is equipped with a motion infrared-ray camera and is used to monitor the heart rate of the player. RFA could estimate the optimal exercise intensity for each player and perform fine-tuned upregulation and downregulation based on this physiological feedback (45). Therefore, providing an appropriate amount of exercise for all generations, from children to the elderly, has become possible (46, 47).

Routinely, the player controls the character by jogging or squatting in the Adventure Mode. Each stage of the Adventure Mode consists of several battle scenes; in addition to aerobics training, the player must perform intensive RT and yoga exercises that exert stress on the muscles of the entire body to defeat the enemy and clear the stage. Players are rewarded based on the amount of exercise they perform and continue to advance while continuously improving their skills. Given that many individuals living in the LTCFs are becoming older and sicker and require more assistance with their ADLs, they have a higher potential risk of falls, and exergame-based exercise should be supervised to reduce unnecessary walking (40). Moreover, some individuals living in LTCFs use wheelchairs mainly for ambulation (28). Therefore, we will use the knee assist mode in RFA, which helps the player character jog and sprint when it is vital for level completion in the game. This means that traversal of the Adventure Mode is possible for players who only have reliable or pain-free use of their arms and trunk.

At the beginning of the game, we will enter the age, sex, and weight of each player, and they should stretch and squeeze the Ring-Con as hard as possible to measure their maximal intensity (this step could be considered the concept of 1 RM as in the traditional RT). Then, the Nintendo Switch sets the initial amount of exercise that must be performed in each stage and adjusts it progressively and automatically depending on the performance of the player. The ideal intensity of each session will set to be 13 (somewhat hard) on the Borg Rating of Perceived Exertion (RPE) scale (48). The higher the intensity, the more repetitions and the larger resistance of the Ring-Con to be stretched and squeezed. The supervisor could also adjust the intensity level manually based on the performance of the player; however, we will not adjust the intensity level manually in this study to ensure the consistency of the protocol. The Joy-Con on the Ring-Con could detect the heart rate of the player after the training session. RFA could provide a summary of the training results, such as exercising time, total calories burned, and total distance ran, after each training session. Warm-up and cool-down will last 10 min each for each training session. In addition to the data RFA records, we will measure the blood pressure, heart rate, heart rate reserve, and oxygen saturation using a pulse oximeter before and after each training session.

As for the skills required to complete the game, in addition to the regular stretching and squeezing that are mandatory for the character player to progress and earn rewards in the Adventure Mode, we have developed six fit skills that focus on training the upper extremities and trunk from the list provided by RFA, including overhead press, shoulder press, front press, bow pull, overhead arm spin, and triceps kickback. The player must choose three of the six aforementioned fit skills to defeat the monster in each beat mode. The flowchart of the exergame-based exercise and the corresponding trained muscle and joint movement is provided in Table 1.

### Contents of standard care (control group)

The standard care in the control group will be applied as usual in our LTCFs, in the way of group activities, including calisthenics (that could be performed in the sitting position), horticultural therapy, and group static activities (e.g., tabletop games). The programs will be performed two times per week for approximately 30–60 min, depending on the activity, and will be led by a therapist.

### Blinding

Because of the design of the study and the nature of the interventions, blinding the staff and participants of the LTCFs is impossible. The assessors, measuring the outcomes, and the interpreter, analyzing the data, will be blinded in this study.

## Outcomes measured

All participants will be subjected to three evaluations. The first evaluation is at baseline (Time 0), which is before randomization. The second and third evaluations will be performed at the end of the 6^th^ (Time 1) and 12^th^ (Time 2) weeks after the intervention, respectively (Table 2).

**TABLE 2:** Instruments and measures to be implemented for data collection.

Instrument or measure	Outcome	Description	Time point	References
Appendicular skeletal muscle mass index	Diagnostic criterion of sarcopenia. Primary outcome	Measured by bioelectrical impedance analysis. Defined as the appendicular skeletal muscle mass (Kg) divided by the height squared (m^2^)	T0, T1, T2	(49, 52)
Dominant handgrip strength	Diagnostic criterion of sarcopenia. Primary outcome	Measured by JAMAR dynamometer under standard position.	T0, T1, T2	(54)
Gait speed	Diagnostic criterion of sarcopenia. Primary outcome	The participants are asked to walk at a normal speed on a 6-m long corridor without a barrier and the usual gait speed calculated by measuring the time spent by a participant.	T0, T1, T2	(52)
Study of osteoporotic fracture index	Indicator of frailty. Primary outcome	Including three components: (a) a weight loss of ≥ 5% during the preceding year, (b) an inability to rise from a chair five times without using the arms, and (c) an answer of “no” to the question “Do you feel full of energy?”	T0, T1, T2	(57)
Box and block test	Indicator of hand dexterity. Secondary outcome	The number of blocks the participants transferred in 60 seconds from one compartment to the other compartment of the wooden box.	T0, T1, T2	(59)
Biceps and triceps brachii muscle strength of the dominant side	Indicator of muscle strength. Secondary outcome	Using the microFET^®^ 3 to measure the maximal voluntary isometric contraction under standard positions.	T0, T1, T2	(62)
Sonographic thickness of biceps and triceps brachii, quadriceps, and gastrocnemius muscles	Indicator of muscle morphology. Secondary outcome	Using a portable LOGIQ e ultrasound, equipped with a 5-12 MHz linear array transducer, to measure the muscle thickness under standard positions.	T0, T1, T2	(63, 64)
Range of motion of the joints of upper extremity	Indicator of functional movement. Secondary outcome	Using a goniometer under standard positions to measure shoulder flexion, abduction, and external rotation; elbow flexion and extension; forearm supination and pronation and wrist flexion and extension.	T0, T1, T2	(65)
Kihon checklist-Taiwan	Indicator of general function. Secondary outcome	A self-reported questionnaire, consisting of 25 items divided into 7 sub-categories. Each item is rated as pass (0) or fail (1). A higher total score indicates a lower level of function.	T0, T1, T2	(66)
Medical outcomes study 36-Item short-form health survey	Indicator of health-related quality of life. Secondary outcome	A self-assessment containing 36 items, divided into 8 subscales. Responses to each question were transformed to a scale ranging from 0–100. The higher the scores, the better the quality of life.	T0, T1, T2	(70)
Brain health test brief cognitive test	Indicator of cognitive function. Secondary outcome	A clinical assessment tool, including orientation to time, immediate and delayed recall of five items, categorical verbal fluency test (listing four-legged animals in one minute), and the Clock Drawing Test. The higher the scores, the better the cognitive function.	T0, T1, T2	(71)

T0, baseline measurement; T1, the end of the 6^th^ week after the exergame-based exercise; T2, the end of the 12^th^ week after the exergame-based exercise.

## Primary outcomes

The primary outcomes chosen for this study are the criteria for the diagnosis of sarcopenia proposed by the AWGS, including appendicular skeletal muscle mass index (ASMMI), dominant HGS, and usual gait speed. Moreover, the Study of Osteoporotic Fractures Index (SOF index), which indicates the frailty status, is also one of the primary outcomes.

### Anthropometry and body composition: Appendicular skeletal muscle mass index

We will perform bioelectrical impedance analysis (BIA) to evaluate the participant’s appendicular skeletal muscle mass. Compared with other established methods for measuring body composition, including dual-energy X-ray absorptiometry, computed tomography, and magnetic resonance imaging, BIA has been widely used in clinical settings because it is relatively simple, quick, and non-invasive (49). BIA of the tetrapolar eight-point electrode type (InBody S10 for the supine measure, InBody Co., South Korea) will be used in this study given that some participants will be difficult to measure in the standing position. This BIA model enables multifrequency impedance measurement of the arms, trunk, and legs using eight electrodes positioned at each hand and foot. Impedance parameters are measured with alternating currents of 80 and 100 mA at frequencies of 1, 5, 50, 250, 500, and 1,000 kHz for InBody S10 (50). The participants will be placed in the lying position for approximately 10–15 min before the test so that body water may be dispersed evenly inside the body. The four extremities should be spread naturally to a 15° angle away from the trunk to ensure that the extremities do not touch the trunk part of the body (51). The device will be calibrated in the morning using a standard control circuit supplied by the manufacturer. The ASMMI is defined as the appendicular skeletal muscle mass (in kg) divided by the height squared (in m^2^) (52).

### Dominant hand grip strength

The HGS will be measured using a JAMAR dynamometer (J A Preston Corporation, New York, NY, USA) using all five notches. A JAMAR dynamometer is a hydraulic instrument that measures isometric strength in kilograms. It has been proven to have good reliability in various older populations (53). The measurement will be performed under a standard position, and the instrument should be freely held. Each participant will be instructed to sit straight with their upper arm in a neutral position and their elbow flexed at 90°C, their forearm in a neutral position, and their wrist at 0–30°C extension (54). The measurement should be performed three times, and the highest of the three measurements will be recorded. The participants will be allowed to rest for 1 min between measurements. The minimal clinically important difference (MCID), defined as the minimal amount of change required to distinguish a true performance change due to variability in performance or measurement error, is also measured in this study. No available studies on the MCID of the HGS in older adults so far, whereas, in healthy individuals, the MCID of the HGS is 2.44–2.69 kg (55).

### Gait speed

The participants will be instructed to walk at a normal speed on a 6-m-long corridor without a barrier, and the usual gait speed is calculated by measuring the time spent to reach the end of the corridor by a participant as suggested by the AWGS (52). The participants could walk with or without assistive devices during the measurement. The time will be initially recorded once the participants start walking and stop at the point when they reach a distance of 6 m. Gait speed should be measured two times, and the average of the two speeds will be recorded. The participants will be allowed to rest for 10 min between measurements. The MCID of gait speed across multiple patient groups is 0.10–0.20 m/s (56).

### Study of osteoporotic fractures index

The SOF index consists of the following three components: (a) a weight loss of ≥5% during the preceding year (regardless of the intention to lose weight), (b) the inability to rise from a chair five times without using the arms, and (c) an answer of “no” to the question “Do you feel full of energy?” The participants will be identified to be frail if they have two or more of the aforementioned components; those with one disability are considered in pre-frailty status, and those with none of the aforementioned impairments are considered robust (57). The SOF index has been proven to be a valid tool to evaluate frailty, particularly for community-dwelling older adults in Taiwan (58).

## Secondary outcomes

### Manual dexterity

The box and block test (BBT) can be used to measure the unilateral gross manual dexterity in various populations with high test–retest reliability and validity (59). The setup of the BBT consists of a wooden box divided into two compartments, with 100 wooden blocks inside one compartment. The participants will be instructed to transfer the wooden blocks one by one from one compartment to the other in the sitting position. The score is based on the number of blocks the participants transferred in 60 s. Most studies on the MCID of the BBT have involved patients with stroke, and the MCID was 5.5 cubes/min for the most affected side and 7.8 cubes/min for the least affected side (60).

### Biceps and triceps brachii muscle strength of the dominant side

We will use the microFET^®^ 3 (Hoggan Health Industries, West Jordan, UT, USA) to measure the maximal voluntary isometric contraction (MVIC) of the biceps and triceps brachii of the dominant side. The microFET^®^ 3 is an electronic handheld dynamometer that can detect 0–150 lb of force with high reliability and validity (61). The participants will be instructed to lie on the treatment table with their elbows forming a 90° angle to the horizontal such that the arm is perpendicular to the limb. The device will be placed on the ventral (for the biceps brachii) or dorsal (for the triceps brachii) side of the arm and aligned with the ulnar styloid. The participants will be encouraged to go against the force, which is exerted toward the device, with their maximum strength (62). The MVIC will be measured two times, and the average of the two measurements is recorded. The participants will be allowed to rest for 1 min between measurements.

### Sonographic thickness of biceps and triceps brachii muscles

A single-experienced operator, who is not involved in any further data analysis and is blinded to clinical symptoms, will use a portable LOGIQ e-ultrasound (General Electric Company, USA, 2010) equipped with a 5–12-MHz linear array transducer to measure the muscle thickness under sonography. Measurements should be performed by gently applying the transducer onto the skin that is coated by a thin layer of water-soluble gel. The transducer should be held orthogonal to the skin surface to ensure precise depth analyses and avoid transmission parallax error. All participants will undergo measurements in the afternoon. All measurements will be performed once on each side and recorded, respectively. The position used to measure each muscle is as follows:

#### Biceps brachii muscle

The thickness of the brachial biceps will be obtained at two-third of the way between the acromion and the antecubital crease of the examined upper limb with the transducer placed perpendicular while exerting minimum pressure. The examined upper limb should be extended fully. The thickness is measured in the transverse plane (63).

#### Triceps brachii muscle

The thickness of the brachial triceps will be obtained at the proximal one-third of the way between the acromion and the olecranon of the examined upper limb with the transducer placed perpendicular while exerting minimum pressure. The examined upper limb should be overhead and extended fully. The thickness is measured in the transverse plane (64).

### Measurement of the range of motion of the joints of the dominant upper extremity

The range of motions (ROMs) of the dominant upper extremity, including shoulder flexion, abduction, and external rotation; elbow flexion and extension; forearm supination and pronation; and wrist flexion and extension, will be measured. The ROMs will be measured using a goniometer under standard positions (65).

### General function

We will use the Kihon checklist-Taiwan (KC-T) to indicate the ADLs of the participants in this study. The KC-T is a self-reported questionnaire consisting of 25 items divided into seven subcategories: general independence, physical strength, nutrition, oral function, level of social activities outside the home, cognitive function, and risk of depression. Each item is rated as a pass (0) or fail (1); therefore, a higher total score indicates a lower level of function (66). The KC-T is used by the Ministry of Health and Welfare in Taiwan as an outcome indicator for community-based programs that delay and prevent disability (67), and its usability in practice in real-world settings in Taiwan has been proven (68).

### Health-related quality of life

We will use the Medical Outcomes Study 36-Item Short-Form Health Survey (SF-36) to indicate the HRQoL of the participants in this study. The SF-36 is a self-assessment validated generic health survey containing 36 items divided into eight subscales: physical functioning, role limitation due to physical problems, bodily pain, general health, vitality, social functioning, role limitation due to emotional problems, and mental health. The first four subscales represent the physical function, and the subsequent four subscales represent the mental function. Responses to each question will be transformed into a scale ranging from 0 to 100. The higher the scores, the better the HRQoL (69). All subscales have high levels of validity and reliability. The SF-36 has an acceptable internal consistency (Cronbach’s α value ranged from 0.74 to 0.95) among the Taiwanese population (70). We will record the scores of the entire questionnaire, physical function, and mental function in this study.

### Cognitive level

We will use the Brain Health Test (BHT)–brief cognitive test (BHT-Cog) to measure the cognitive level of the participants in this study. The BHT, developed by the Taiwan Dementia Society, is a simple dementia screening tool with high validity that can help primary care physicians identify patients with cognitive impairment among subjects with memory complaints or those at a high risk of dementia. It consists of risk factors and a brief cognitive test. The BHT-Cog includes orientation to time, immediate and delayed recall of five items, a categorical verbal fluency test (listing four-legged animals in 1 min), and the Clock Drawing Test (10:10) (71). The Ministry of Health and Welfare also uses the BHT in Taiwan as an outcome indicator for community-based programs that delay and prevent disability (67).

## Statistical analysis

### Sample size calculation

Since a high-quality study evaluating the effects of a similar intervention on muscle strength, physical activity, and function was unavailable when we wrote our protocol, we used previous results from one study using augmented reality (AR)-based exercise for one of our primary outcome measures (ASMMI) (72) for this power calculation. The effect size of a similar study for the AR-based intervention is high (0.71). Based on G*Power (version 3.1.9.2, for Windows), at least 18 observations in each group should be recruited by detecting a difference of 2 standard deviations (SD) of the ASMMI between the groups with a power of 80% and alpha of 5%, and the effect size is determined to be high (*F*-test family, 0.4) (73). To account for an expected dropout rate of 40%, given that most participants in the LTCFs have comorbidities, we decided to increase this number to a group size of 26. Thus, the aim will be to include at least 52 patients in the study.

### Quantitative data

We will use Statistical Package for the Social Sciences for Windows (version 19.0; Released 2010; IBM Corp., Armonk, NY, USA) for all statistical analyses. Continuous data are expressed as means with SDs, and categorical variables are presented as absolute numbers or percentages. The normality and homoscedasticity of the data will be checked before each analysis. For comparisons of demographic data between the experimental and control groups, the chi-square test, independent *t*-test, and Mann–Whitney *U*-test will be used as appropriate depending on the features of distributions of the data. As for the training effects on outcomes, a mixed analysis of variance, with time as a within-subject factor and intervention as a between-subject factor, will be performed. *Post hoc* analysis will be performed using the Bonferroni test. *P*-values < 0.05 will be used to indicate statistical significance. If both the data and the residuals are not normally distributed for ANOVA, we will use bootstrapping to get confidence intervals and use those to determine whether effects are statistically significant, rather than using *p*-values directly.

## Anticipated results and discussion

This study protocol describes the design of an RCT that evaluates the clinical effect of the exergame-based exercise delivered *via* RFA among older adults in LTCFs. Individuals around the world live longer nowadays, and many countries will become a super-aged society soon. Preventing and delaying the loss of intrinsic capacity and functional ability of older adults and helping them age successfully are crucial for us (74). Elderly individuals living in LTCFs are more prone to geriatric syndromes, such as frailty and sarcopenia, than those living in the community. Moreover, many LTCFs lack healthcare professionals, particularly those in rural regions. This project aims to study whether we can use exergame-based exercise as an alternative to previous manpower-consuming therapies in LTCFs. Given that the concepts of frailty and sarcopenia are multifactorial and their definitions vary, this study focuses on the parameters of muscle and functional performance.

This project is unique as the exergame-based exercise is delivered *via* RFA. Similar to other exergames, RFA uses a gamified approach and immersive scenarios to motivate the player by role-playing (31). It could provide both visual feedback from the screen and sensory feedback from the Ring-Con. Apart from other exergames, with its featured Ring-Con, RFA could be considered a multicomponent exercise, combining PRT and aerobic exercises for strength, balance, and muscle stretching. Studies have evaluated the effectiveness of PRT among community-dwelling older adults and proven that it is easily available at a low cost and effective in improving physical function and strength (10, 75). Therefore, we are looking forward to the clinical effectiveness in improving the muscle parameters after this study.

Another feature of using RFA to deliver exergames is that it could set up the initial amount of exercise that must be performed in each stage and adjust it progressively and automatically depending on the performance of the player. Therefore, the progression for the exercise prescription might be individualized, and it is time- and manpower-saving given that it could be modified by the machine. The ACSM suggests increasing intensity over time to maintain the intensity of the exercises at moderate levels (41–60% of 1 RM for resistance exercise and Borg RPE 12–14 for aerobic exercise) (76). Therefore, the study protocol sets the target intensity of the exergame-based exercise at Borg RPE 13 and leaves the weight intensity to Nintendo Switch itself. Although it is practical to do so in real-world settings, and it is safer for the elderly to perform PRT by squeezing or stretching the Ring-Con, one limitation is that we cannot confirm whether the weight intensity given by the Ring-Con is sufficient at moderate levels.

Given that many elderly individuals in LTCFs use a wheelchair for community ambulation because of poor muscle endurance even though they can walk for a short distance to complete gait measurements (40), the exergame-based exercise of this project focuses on training the upper extremities and trunk. By doing so, the participants could be trained in the sitting position to avoid the potential risk of falls. Therefore, most outcomes of this project, including the HGS, MVIC, sonographic thickness of the biceps and triceps brachii muscles, ROMs of the joints of the dominant upper extremity, and BBT, are measured to evaluate the training effects. A study has proven that aging can attenuate the hypertrophic response of muscle groups to RT (77). Contrary to the findings from healthy young adults, in whom neural factors account for a larger proportion of the initial strength increment and muscle hypertrophy becomes the dominant factor after the first 3–5 weeks (78), the effect of muscle training may entirely depend on the neuromuscular adaptation among older adults after an 8-week training course (79). Given the results from the classical studies by Moritani and deVries and considering that the project lasted only 3 months, we measured not only the MVIC but also the sonographic thickness to see whether some early morphological changes in the trained muscles could be detected earlier.

The primary outcomes in this protocol are the criteria to diagnose frailty and sarcopenia. Therefore, this project will measure the gait speed through the exergame-based exercise, which focuses on the upper extremities and trunk only. Walking is a complex movement that requires several functional tasks, such as ROMs, velocity, position, and trained muscles (80). The participants must balance in different positions, such as leaning forward, forward reaching, side shuffle, and lateral shifting, to use the RFA. Those movements might occur at the trunk/hips/knees, although the protocol does not allow the performance of PRT on the lower extremities. Granacher et al. demonstrated that the ability of older adults to rise from a chair, ambulate, and make turns improves after 9 weeks of core muscle strength training (81). Park et al. observed that walking speed increased after a sitting boxing program focusing on upper extremity stretching and strengthening for 6 weeks (82). Both studies have proven that strengthening programs can also induce adaptive processes, particularly in the neuromuscular system, thereby enhancing balance performance and functional mobility. Moreover, interlimb and intralimb segment coordination are important for bipedal human gait. Arm swing in the human gait cycle plays an active role in maintaining body posture. The gait speed increases when the amplitude of the arm and leg is increased (83). Given the aforementioned three main reasons, the exergame-based exercise in this study might increase gait speed.

By presenting with heterogeneity, one recent systematic review with the recruitment of 15 eligible RCTs found that exercise not only has a positive effect on physical outcomes but also improves QoL and ADLs in elderly individuals (84). Therefore, in addition to measuring the clinical effects of the exergame-based exercise on parameters of muscle and functional performance, this protocol also includes the QoL and ADL as secondary outcomes. The SF-36 is used to indicate the HRQoL because it is a validated self-assessment generic health survey with a reliable intraclass correlation coefficient (70). KC-T will be used to indicate the ADL since its usability in practice in real-world settings in Taiwan has been proven (68), and it is widely used in LTCFs. Moreover, both the SF-36 and Kihon checklist have Taiwanese versions, which are important when measuring the QoL and ADL because the values toward QoL and ADL vary from culture to culture.

No cutoff value of available cognitive measurements allows us to screen who is suitable for exergames. Most studies on exergames in the elderly recruited participants with sufficient cognition levels to understand the orders and procedures required in the game (36). Many older adults in LTCFs have some cognitive deficits, though the degree varies in Taiwan (68). Exergames have therapeutic effects on cognition among older adults (34, 36). Although the manner of play varies among exergames, there are some commonly shared concepts. Exergame environments provide an extra spatial feature. Players confront challenging tasks with visual and auditory stimuli, cues, and feedback. Immersion into a virtual environment also redirects the player’s experiences to improve attention restoration, reduce stress, and promote cognitive rehabilitation. Furthermore, exergames require players to make decisions during the game. Exergames delivered *via* RFA provide all aforementioned features, and we expect that the cognitive level of the elderly in LTCFs improves after the training.

Supervised exercise is safe for frail older adults (85). Only one well-trained assistant will be required to supervise the entire exergame-based exercise procedure in this project. Many participants can play the game simultaneously if the number of Nintendo Switch and RFA is sufficient, which is time- and manpower-consuming. However, this study protocol has some limitations. First, this study used the convenience sampling method because recruiting older adults who regularly participate in LTCFs publicly is difficult and impractical. This project might be less representative, even though the minimum estimated sample size can be met. The results of this study should be applied with caution and could only be generalizable to similar populations. Second, the exergame-based exercise and outcome measures only lasted 3 months. This duration might be insufficient to observe an increase in the muscle mass of older patients. However, given that an intervention period of more than 3 months might lead to a higher attrition rate of the participants, particularly for those in LTCFs with several comorbidities, we will also measure muscle morphology using sonography to detect any earlier change in muscle parameters, and we still suggest a 3-month protocol. Third, to our knowledge, this project is the first study to evaluate the effectiveness of using RFA among elderly individuals living in LTCFs. No established protocol for training with RFA could be followed. Although the entire exercise prescription is based on the guidelines for older adults by the ACSM, we cannot ensure that the protocol for the current exergame-based exercise is ideal. Fourth, individualized setting and progression of the exercises automatically are the features of using RFA to train elderly individuals. However, this project can only measure the exercise intensity by RPE. The investigators cannot confirm the exact resistance provided by the Ring-Con during each training session.

If the findings of this study show that the exergame-based exercise *via* RFA could improve the parameters of muscle and functional performance, the training method should be implemented as a treatment option for all older individuals living in LTCFs, particularly for those in rural regions with lesser healthcare resources. A successful exergame-based exercise would provide benefits beyond muscle strength, muscle mass, and ADLs of the elderly because it would also enhance the HRQoL or even help the elderly age gracefully. We expect that the protocol will be useful and practical to implement in real-world settings. With the positive results of this protocol, further larger studies could be conducted to examine whether the exergame-based exercise *via* RFA could be a treatment alternative to time- and manpower-consuming therapies currently applied in LTCFs.

## Ethics statement

Written informed consent was obtained from the individual for the publication of any potentially identifiable images or data included in this article.

## Author contributions

S-HT and Y-JT planned the project and developed the research design. S-HT and S-FS calculated the sample size. L-HC and K-LL were responsible for literature reviewing and evaluating the appropriate measurement tools. S-HT wrote the first draft. Y-JT, L-HC, and S-FS were responsible for the revisions of the manuscript. All authors read and approved the final manuscript version.
